# Genetic therapies for neurological disorders

**DOI:** 10.1007/s00439-021-02399-5

**Published:** 2021-11-22

**Authors:** Laura FitzPatrick, Adrian Bird

**Affiliations:** Wellcome Centre for Cell Biology, University of Edinburgh, The Michael Swann Building, Max Born Crescent, Edinburgh, EH9 3BF UK

## Abstract

In recent years, it has become increasingly apparent that many neurological disorders are underpinned by a genetic aetiology. This has resulted in considerable efforts to develop therapeutic strategies which can treat the disease-causing mutation, either by supplying a functional copy of the mutated gene or editing the genomic sequence. In this review, we will discuss the main genetic strategies which are currently being explored for the treatment of monogenic neurological disorders, as well as some of the challenges they face. In addition, we will address some of the ethical difficulties which may arise.

## Introduction

Recent advances in DNA sequencing technology have led to an explosion of knowledge about the genetics of human disease, and the realisation that many more disorders are genetic in origin than previously thought. For example, large-scale exome-sequencing projects, such as the Deciphering Developmental Disorders study, have identified novel pathogenic de novo mutations in patients with undiagnosed neurodevelopmental conditions (Fitzgerald et al. 2015; McRae et al. 2017). The new knowledge has led to a surge of interest in the potential for therapies which address the genetic root cause of these disorders, rather than attempting to treat secondary consequences. These approaches include conventional gene therapy (also referred to as “gene transfer”) which aims to restore function of the mutated gene by introducing a functional copy into cells (Friedmann and Roblin 1972). In addition, advances in our ability to re-write DNA sequences via genome editing, particularly “clustered regularly interspaced short palindromic repeats” (CRISPR) technology, have sparked interest in their use for the treatment of a variety of disorders. All of these approaches are particularly suited to monogenic conditions which, in theory, can be cured by correction of the disease-causing mutation. In this review, we illustrate the growing therapeutic potential of these developing technologies. We also consider the technical challenges still to be overcome, as well as some ethical issues posed by genetic interventions in the brain.

Like many medical innovations, genetic therapies rely on basic knowledge acquired in model organisms. Importantly, there needs to be evidence that symptoms have the potential to be alleviated or even cured. A disorder which highlights the value of pre-clinical research is Rett syndrome (RTT), a severe neurological disorder caused by mutations in the X-linked gene *MECP2* (Amir et al. 1999). Mouse models recapitulate many features of the human condition (Chen et al. 2001; Guy et al. 2001), supporting the conclusion that the function of the MeCP2 protein is the same in mice as in humans. Importantly, the majority of symptoms can be reversed in adult *Mecp2*-null mice by restoring expression of the wild-type protein (Guy et al. 2007; Robinson et al. 2012). This suggests that RTT is curable well after the onset of symptoms. Phenotypic reversibility of a few other monogenic neurological conditions has been tested using mouse models, with variable results. For example, restoration of *Ube3a* expression in young mice leads to reversal of many adverse phenotypes in a model of Angelman syndrome, but not all autism-related phenotypes are reversed when the gene is activated in older animals (Silva-Santos et al. 2015). These findings point to an early window for therapeutic intervention. In all neurodevelopmental disorders of this type, basic understanding of the function of the mutated gene and its time of action during life is an important pre-requisite for intervention.

## What genetic interventions are under development?

### Gene transfer

The technically most straightforward approach to genetic therapy involves introduction of wild-type copies of the affected gene into the nucleus of appropriate cells in the brain (Friedmann and Roblin 1972). To successfully achieve this, a suitable delivery vehicle which can efficiently bring genetic material to the target cells is required. At present, adeno-associated viral (AAV) vectors are the preferred delivery vehicle for the central nervous system (CNS) due to their relatively favourable safety profile and the potential of certain serotypes (such as AAV9) to cross the blood–brain barrier, infect post-mitotic neurons and provide widespread and sustained transgene expression throughout the brain (reviewed in Hudry and Vandenberghe 2019). The advantages and disadvantages of AAV vectors will be considered in more detail later.

The success of gene transfer has been demonstrated for several genetic disorders, and AAV-based therapies have been clinically approved for use in humans, including Glybera and Luxturna for the treatment of lipoprotein lipase deficiency and *RPE65* mutation-associated inherited retinal dystrophy, respectively (reviewed in Ginn et al. 2018). More recently, in 2019, Zolgensma was approved for the treatment of spinal muscular atrophy (SMA). Introduction of a functional *SMN1* (survival motor neuron 1) gene into motor neuron cells effectively prevents fatal loss of muscle function in children with SMA (Mendell et al. 2017).

### Genome editing using programmable nucleases

An ideal genetic therapy would correct the mutated gene by “re-writing” its DNA sequence to rectify the mistake. Not only does this address the root cause of the problem, but its effect would be permanent. Initial approaches have involved fusing DNA binding domains that can target a specific genomic location with high accuracy, to nucleases, which generate double-stranded breaks (DSBs) in the DNA (reviewed in Cox et al. 2015). These include the CRISPR/Cas9 system, which is adapted from a bacterial immune system, and involves targeting of the Cas9 nuclease to sites of interest via guide RNAs (Gasiunas et al. 2012; Jinek et al. 2012). What happens next is highly dependent on how the break is repaired by the cell, which can vary depending on factors such as the cell type/state and availability of a repair template (reviewed in Ceccaldi et al. 2016).

Precise changes can be introduced into the genome using CRISPR/Cas9 if a homologous DNA repair template is supplied at the same time, permitting repair by the high-fidelity homology-directed repair (HDR) pathway (Cong et al. 2013; Paquet et al. 2016). Unfortunately, the therapeutic potential of HDR-based approaches for in vivo genome editing approaches is limited by their inefficiency in human cells (Mao et al. 2008). Instead, the predominant repair pathway for DSBs in mammalian cells, including post-mitotic neurons, is non-homologous end-joining (NHEJ), which generates a heterogeneous range of repair products (Rothkamm et al. 2003; Ren and Peña De Ortiz, 2002; reviewed in Chang et al. 2017). It was previously thought that repair outcomes were random and were therefore of limited therapeutic use beyond gene disruption. However, recently it has been shown that consequences for a given target site are highly reproducible and can often be accurately predicted (van Overbeek et al. 2016; Allen et al. 2018; Shen et al. 2018; Chakrabarti et al. 2019). The potential of predictable template-free CRISPR/Cas9 editing has been demonstrated for pathogenic frameshift mutations, for example, by identifying targets which predominantly restore the wild-type reading frame (Shen et al. 2018). Other possibilities for therapeutic genome editing using programmable nucleases are discussed in Wang et al. (2020).

### Direct base-editing

This highly desirable option seemed technically beyond reach until a recent breakthrough came with the development of programmable DNA base editors, which can change base pairs without requiring DSBs, homology-directed repair or donor DNA templates (Komor et al. 2016; Gaudelli et al. 2017). Base editors typically consist of a catalytically impaired Cas9 fused to cytidine—or adenosine—deaminase enzymes (reviewed in Porto et al. 2020). These artificial proteins can be targeted to disease-causing mutations and potentially revert them to the wild-type sequence, offering permanent correction in a single-dose. “Prime editing” is another exciting development, which involves catalytically impaired Cas9 fused to an engineered reverse transcriptase, and has the potential to correct a broad range of mutations (Anzalone et al. 2019).

### RNA editing

Instead of editing the DNA, an alternative strategy is to correct its messenger RNA (mRNA) transcripts. Programmable RNA editors have been developed which fuse domains from endogenous RNA editing enzymes, for example the deaminase domain of RNA-specific adenosine deaminases, to the RNA binding domain from a different protein (Montiel-Gonzalez et al. 2013; Cox et al. 2017). Engineered guide RNAs are then introduced to recruit the RNA editor to its target sequence in the mutant mRNA. In the case of RTT, this approach has the theoretical potential to correct at least 36% of disease-causing mutations by adenosine-to-inosine editing (Sinnamon et al. 2020). Introduction of RNA editors into the hippocampus of *Mecp2*-mutant mice corrected ~50% of mRNA transcripts and restored MeCP2 function to a similar level (Sinnamon et al. 2020). This is likely to be enough for significant phenotypic improvement in mouse disease models, although this is yet to be experimentally tested.

## The challenge of delivery

As mentioned above, AAV-mediated delivery of components leads to widespread expression of protein throughout the brain (Foust et al. 2009; Chan et al. 2017). An important benefit is that expression of the cargo gene is sustained for months or years in non-dividing cells. Transgene expression has been reported to persist in neurons for more than 4 years in humans (Mittermeyer et al. 2011) and at least 15 years in non-human primates (Sehara et al. 2017). This is a critical advantage for gene transfer strategies because it allows them to provide long-term correction without the need for re-administration.

### Issues with gene dosage

Despite the attractions of AAV vectors, they have limitations. An important reservation is lack of control over the number of virus particles delivered per cell. This is of particular concern where the level of the therapeutic agent needs to be tightly regulated. For example, while insufficient MeCP2 leads to RTT, too much MeCP2 is associated with the severe neurological disorder “MeCP2 duplication syndrome” (Collins et al. 2004; Luikenhuis et al. 2004; Van Esch et al. 2005). Accordingly, toxicity due to MeCP2 overexpression has been observed in preclinical gene transfer studies in wild-type animals (Gadalla et al. 2017; Sinnett et al. 2017). Furthermore, in female RTT patients half of the cells express normal levels of MeCP2, which means that indiscriminate delivery of AAV vectors to these cells may lead to overexpression-mediated toxicity. Another example of a dosage-sensitive gene is *UBE3A*, whose mutation can cause either Angelman syndrome or non-syndromic autism as a result of loss-of-function or overexpression, respectively (reviewed in Khatri and Man 2019). To counter this problem, the search is on for regulatory elements that could be included within the delivery vector, although packaging constraints mean that it is often difficult to fit all necessary nucleic acid sequences within a single virus.

An important advantage of Cas9 nucleases and DNA/RNA editors is that they avoid deleterious over-expression of this kind since the gene remains under the control of its natural regulatory elements. Here again, however, the large size of these genome editing proteins makes AAV-mediated delivery a challenge. Solutions to this problem include use of smaller variants of Cas9 or splitting the transgene into two segments which automatically fuse together upon expression in the cell (reviewed in Wang et al. 2020). Encouragingly, direct injection of dual AAV vectors expressing two halves of a split base editor achieved base editing efficiencies of up to 59% in the mouse cortex (Levy et al. 2020).

### Transduction efficiency

For genetic diseases affecting the brain, the low level of neuronal infection by available AAV serotypes is also a critically important issue. Although increasing the dose of AAV9 vectors enhances brain transduction, this can lead to a concomitant increase in transduction of peripheral tissues, such as the liver and heart (Gadalla et al. 2013, 2017). Importantly, high doses of AAV vectors should be avoided, as this can lead to severe toxicity, including death, in both non-human primates (Hinderer et al. 2018; Hordeaux et al. 2018) and humans (Wilson and Flotte, 2020). While engineered AAV variants can infect the majority of neurons in specific strains of mice (Deverman et al. 2016; Chan et al. 2017; Hordeaux et al. 2018; Huang et al. 2019), serotypes that can achieve similar levels in non-human primates have not yet been reported. Consequently, therapies that depend on the delivery of DNA to most neurons throughout the brain await improvements in vector infectivity.

In spite of the relatively low efficiency of AAV transduction, it remains possible that transduction of a small proportion of neurons may nevertheless yield significant therapeutic benefit. In the case of RTT, for example, reversal experiments and gene therapy studies in *Mecp2*-null mice have given encouraging results. Activation of *Mecp2* expression in ~ 70 to 80% of cells in the brain led to an impressive reversal of most RTT-like symptoms in *Mecp2*-null mice (Guy et al. 2007; Robinson et al. 2012), but also transduction of 10–40% of cells by direct brain injection with AAV-*MECP2* vectors improved RTT-like symptoms and greatly extended survival (Gadalla et al. 2017). Even transduction efficiencies as low as 3–5% significantly improved survival of adult *Mecp2*-null mice (Gadalla et al. 2017). These results suggest that desirable symptomatic improvements may result even if only a small proportion of brain cells have MeCP2 expression restored.

### Immunogenicity

Another challenge is the potential for foreign transgenes (e.g. bacterially derived Cas9), to stimulate host immune responses. Long-term expression of CRISPR/Cas9 components was generally well tolerated in a mouse model of muscular dystrophy after one year, although a host immune response was detected in nearly all mice injected as adults (Nelson et al. 2019). The prevalence of pre-existing immunity to AAV capsids (Calcedo et al. 2009; Kay, 2011; Mingozzi and High, 2013) and Cas9 nucleases (Charlesworth et al. 2019; Wagner et al. 2019) in humans is another concern. Current engineered capsid variants may not be different enough from naturally occurring AAV serotypes to evade detection by neutralising antibodies, although efforts are underway to develop highly diverse variants to counter this problem (Bryant et al. 2021). Direct administration to the immune-privileged brain could potentially mitigate these problems, as might immunosuppressive strategies (reviewed in Perez et al. 2020).

### Genotoxicity

Off-target editing is one of the primary safety concerns for the use of genome editors (nuclease-based or DNA base editors), since undesirable changes could be permanently introduced into the genome (reviewed in Doudna 2020). While sustained expression of AAV vectors is an advantage for gene transfer strategies, prolonged expression of CRISPR/Cas9 components may be detrimental as it increases the likelihood of off-target editing (Zuris et al. 2014; Ishida et al. 2015). This effect may be mitigated by engineering high-fidelity variants and/or minimising the duration of Cas9 expression (reviewed in Zhuo et al. 2021). Alternatively, non-viral delivery vehicles, such as nanoparticles, could be used. Non-viral delivery of CRISPR/Cas9 components can mediate localised genome editing in the brain close to the administration site (Staahl et al. 2017; Lee et al. 2018), but a drawback is that they are unable at present to provide widespread expression throughout the brain.

An additional concern for AAV-mediated delivery of programmable nucleases is accumulating evidence that the introduction of DSBs into the genome during editing can lead to integration of the AAV vector into the host genome (Miller et al. 2003, 2004; Anguela et al. 2013; Hanlon et al. 2019; Nelson et al. 2019). This problem is largely avoided by the use of base editors, which do not tend to generate DSBs in the DNA (reviewed in Anzalone et al. 2020). This represents a further advantage of the base editing approach.

## Efficacy and cost

A critical question for all genetic therapies concerns efficacy. Will the intervention reverse or significantly ameliorate symptoms on a long-term basis without causing undesirable side effects? Even if animal experiments lead us to the strong conclusion that the answer to this question is ‘yes’, the leap to humans is inevitably accompanied by risk. Due to this uncertainty, arguments for or against genetic therapies ebb and flow. An example is the proposal that development of a “cure” for severe neurological disorders may in fact be undesirable from several perspectives (Clarke and Abdala Sheikh 2018). For example, in the context of RTT it was noted that rapid activation of *Mecp2* expression in mice resulted in toxicity and lethality within a few days (Guy et al. 2007). Sudden re-expression of MeCP2 in humans, it was suggested, may lead to autonomic instability (Clarke and Abdala Sheikh 2018). However, due to the low transduction efficiency of current delivery vectors, it is unlikely that *MECP2* will be corrected in a sufficient number of neurons to cause toxicity. Other highlighted uncertainties persist, however, including the impact of changes in brain volume and the psychological adjustments that would accompany improvements in brain function. For this and other genetic disorders of the brain, detailed pre-clinical research has the potential to minimise the chances of an adverse response, but only application of the therapeutic technology in the clinic can fully assess outcomes. In the case of RTT, preclinical studies have confirmed that gene transfer is a promising therapeutic strategy for RTT. Accordingly, several candidates are currently under development in academic programmes and commercially, aiming towards clinical trials within the next few years.

A serious caveat to genetic therapies is their high cost. For example, Zolgensma for the treatment of spinal muscular atrophy has been dubbed the “most expensive drug in the world” costing more than $2 million in the US. This means that in countries without national healthcare programmes, these therapeutics are only available to the privileged. The cost of treatment has been defended by claims that it should be measured against the lifetime of healthcare treatment that should no longer be needed (or at least not to the same extent). Based on precedent, it seems likely that costs will decrease as treatments of this kind become more routine, but given the rarity of some such disorders, bespoke treatments seem destined to always be expensive. Social justice requires that novel therapies that turn out to be effective should be available to all, regardless of socio-economic status. Accordingly, the World Health Organisation (WHO 2021) has recognised the importance of establishing global guidelines to help ensure equitable access to genome editing technologies. However, this will remain a challenging issue and it is likely that different payment strategies will be required, as well as international partnerships to ensure access in low- and middle-income countries. Scientists can also play a role here, as illustrated by the insistence by the Oxford group that the AstraZeneca SARS-CoV-2 vaccine was distributed on a not-for-profit basis.

## Ethical concerns

Editing in germline cells raises long-standing ethical challenges and is currently illegal, so genome editing must be confined to somatic cells. Fortunately, this is technically straightforward, as any modifications introduced into the brain genome will only affect the treated individual and cannot be passed to future generations. Needless to say, rigorous testing will be required to address safety issues and minimise the possibility of unexpected consequences (Nuffield Council on Bioethics 2016; National Academy of Sciences 2020). The need for statutory guidelines has been highlighted by a controversial study which claimed to edit the *CCR5* gene in human embryos, before subsequently implanting edited embryos into two women (commented on in Cyranoski 2019). The aim of the procedure was to disable the *CCR5* gene in order to protect the recipients from certain strains of HIV infection, but the potential for unwanted consequences, including off-target edits or increased susceptibility to other diseases, such as West Nile Virus, were inadequately investigated, making this a high-risk intervention. In light of this scandal, a WHO advisory committee was developed to establish international guidelines for human genome editing. It will be critical that individual scientists, and the scientific community as a whole, adhere to guidelines and ethical regulations set by regulatory bodies, to protect patients, as well as scientific progress.

## Concluding remarks

We are in the midst of a technological revolution which brings closer the possibility of correcting genetic diseases. If the technology brings the promised benefits and alleviates suffering, ethical opposition to manipulation of genes may wane. As with all new medical procedures, however, these advances cannot be achieved without risk. Willingness to undertake this risk is dependent on the severity of the relevant disorder, but also on the attitudes of those concerned, including parents/carers and society at large. Unfortunately, as in the case of organ transplants and many other aspects of medicine in times past, the first usage in humans is fraught with uncertainty. This has been highlighted by the recent fatalities in the Audentes Therapeutics’ AAV gene therapy trial (AT132) for the treatment of X-linked myotubular myopathy (Harrison 2020). It is imperative that there is full transparency within the scientific community, particularly with regard to reporting adverse effects, to ensure that treatments are both safe and effective. However, it is worth noting that the risks of no treatment are also very high for severe neurological disorders and, if successful, patients (and their families) will reap the rewards of a life-changing therapy for these highly debilitating disorders. While a risk-averse (and litigious) society may hesitate at the prospect of the unknown, new therapeutic horizons will never be explored by stasis.

## Data Availability

Not applicable.
